# Effectiveness and safety of endoscopy for treatment of surgical site infection: A randomized control trial

**DOI:** 10.3892/etm.2014.2028

**Published:** 2014-10-17

**Authors:** HAILONG WANG, XINLI DOU, XIANGPING HU, JINSHENG YU, SHAOSHAN WANG

**Affiliations:** 1 Graduate School of Tianjin Medical University, Tianjin 300270, P.R. China; 2 Department of Oncology, Tianjin Binhai New Area Dagang Hospital, Tianjin 300270, P.R. China

**Keywords:** endoscopy, surgical site infection, treatment, randomized trial

## Abstract

The aim of this randomized control study was to evaluate the effectiveness and safety of endoscopy for the treatment of surgical site infection (SSI), compared with conventional therapy. One hundred and six patients who were diagnosed with severe SSI were included in the study, performed from May 2005 to July 2012 at Tianjin Binhai New Area Dagang Hospital, China. Patients were randomly divided into two groups: 57 patients in group A treated by endoscopy and 49 patients in group B treated by conventional therapy for SSI. The primary outcome was the healing period of the wound; the secondary outcomes were the blood loss following surgery, visual analog scale (VAS) measurement, volume of irrigation saline during surgery, rate of skin transplantation, length of hospital stay and other complications. The mean wound healing time was significantly less in group A (10.0±2.5 days) than in group B (19.4±5.2 days). The mean VAS score 7 days after surgery in group A was significantly less compared with group B. The intra-operative blood loss, intra-operative volume of irrigation saline and length of the hospital stay were significantly reduced in group A compared with group B. No significant differences between the groups were revealed in terms of the duration of surgery and the clinical complications. This study demonstrated that the endoscopy procedure for the treatment of SSI reduces the wound healing time compared with that of traditional surgery, without increasing any risk of clinical events. The present study showed that endoscopy was not only effective but also safe in the therapy of serious SSI. However, a further randomized control trial is necessary to testify our conclusions.

## Introduction

Infections that occur in the wound created by an invasive surgical procedure are generally referred to as surgical site infections (SSIs). SSI is one of the most common and serious postoperative complications, occurring in up to 40% of patients undergoing abdominal surgery and 5% of all patients undergoing other surgery, depending on the degree of contamination (1,2). SSI is one of the most significant causes of healthcare-associated infection, and may prolong hospitalization by 5–20 days and substantially increase the cost of healthcare (3–4). It is associated with substantial morbidity and it has been reported that over one-third of postoperative mortality is associated, at least in part, with serious SSI (5).

However, prevalence studies tend to underestimate SSI, since the majority of these infections occur after the patient has been discharged from hospital. It should be noted that the diagnosis covers a large variety of clinical conditions ranging from a relatively trivial wound discharge with no other complications to a life-threatening condition. Other complications of SSI include scars that are cosmetically unacceptable, such as keloid scars, itching, persistent pain and a significant impact on emotional wellbeing (6).

Recent national guidelines concerning the prevention and treatment of SSI have been issued by the National Institute for Health and Clinical Excellence of the UK (7). The poor clinical outcomes of SSI require extra nursing care, intervention and re-operation, and consequently have a high direct or indirect cost. However, various strategies for the treatment of SSI have been tested and employed in the clinic, and the consequences of these treatments have not always been satisfactory for patients or surgeons (3,4). A persistent effort has been made to find a more effective and safer method to treat SSI, such as debridement or drainage (4,6). In this study, we conduct a randomized control trial to evaluate the effectiveness and safety of endoscopy for the treatment of SSI compared with conventional surgery therapy.

## Materials and methods

### Patients

From May 2005 to June 2012, we enrolled patients with different types of SSI at the Tianjin Binhai New Area Dagang Hospital (Tianjin, China) in a prospective, randomized trial to compare clinical results and complications between the endoscopy procedure and traditional open surgery for the debriding of the infected wound.

The inclusion criteria were as follows: SSI patients i) who had undergone surgery >30 days ago; and ii) in which non-surgical treatment was ineffective after SSI. The exclusion criteria were as follows: i) <18 years old; ii) immune system disorder-related disease; and iii) prosthetic implant in the surgical site. Full ethical approval was obtained from Tianjin Binhai New Area Dagang Hospital and all participants provided written informed consent.

To match the two groups, randomization was stratified according to the body regions of the primary surgery (Table I). Subsequently, randomization was performed when the patient was in the anesthetic room immediately before surgery by using the sealed envelope method. The surgical team in theatre were aware of group allocation, but only following the induction of anesthesia. Information on group allocation was not recorded in the clinical or surgical notes, and clinicians undertaking the follow-up of postoperative wounds were fully blinded to the group assignments.

### Interventions (surgical techniques)

In the endoscopy procedure group (group A), the surgical field was sterilized conventionally using sterile towels. A section of the wound approximately 6 mm long was opened along the original incision to permit the entry of the choledochoscope (Olympus T20, Tokyo, Japan). The wound was washed and cleaned with sterile saline. The necrotic tissue and infected suture were completely removed under visualization. For sinuses too small to accommodate the choledochoscope, the necrosis was removed by biopsy clip. Instead of inserting a drainage tube for several days, a saline gauze was used to drain the wound for no longer than 24 h

In the traditional surgery group (group B), the surgical field was sterilized conventionally, as in group A, using sterile towels. Subsequently, the wound was opened through the original incision, washed and cleaned with sterile saline, then drained adequately via negative pressure drainage. A suture was performed 2 days later. In both groups, an antibiotic prophylaxis was administered intravenously 30 min before the surgical incision.

### Outcome measures

The primary outcome of interest was the wound healing time. The secondary outcomes of interest were duration of surgery, blood loss, pain level 7 days after surgery [measured by the visual analog scale (VAS)], volume of irrigation saline, rate of skin transplantation and length of hospital stay.

### Statistical analysis

All analyses were performed using SPSS statistical software (version 17.0). Student’s t-test and the χ^2^ test were performed to analyze the data and determine whether there were differences between the two groups. P<0.05 was considered to indicate a statistically significant difference.

## Results

### Study participants

From May 2005 to June 2012, we enrolled 106 patients with serious SSI in our study and randomized 57 to the endoscopy procedure group (group A) and 49 to the traditional surgery group (group B). No patients were lost during the follow-up period. Patients’ baseline demographical characteristics (age and gender ratio), primary surgery characteristics and size of the infected sites did not differ substantially between the two groups (Table I).

### Outcomes

**Figure 1:**
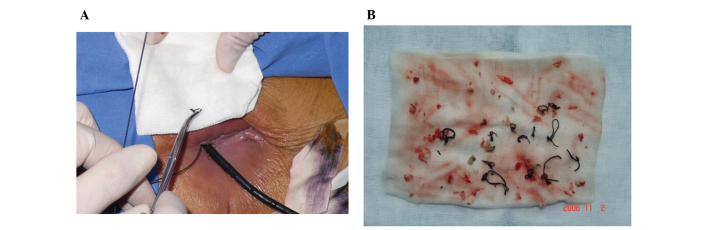
One patient in group A repeatedly experienced surgical wound infection in the perineum following Mile surgery (a surgical procedure for colorectal surgery) for colorectal cancer. The wound had not healed 60 days after the first surgery. The necrotic tissue and infected suture were completely removed during visualization under endoscopy. The SSI wound healed successfully following endoscopic surgery. The images show (A) multiple sutures and (B) necrotic tissue from the sinus.

No significant difference between the groups was noted with respect to the duration of surgery (P=0.06). Three patients in group A (Fig. 1) and six in group B required a skin transplant for skin defects, but the rate of skin transplantation was not statistically different between the two groups (P=0.21; Table II). Patients in group A had a mean intra-operative blood loss of 327±89 ml, which was significantly reduced [mean difference (MD), −78.00; 95% CI, −132.68 to −23.32; P=0.005] when compared with that of group B (405±177 ml). The intra-operative volume of irrigation saline was lower in group A compared with that in group B, and the difference was statistically significant (MD, −770.00; 95% CI, −1082.82 to −457.18; P<0.00001).

The mean wound healing time was also significantly shorter (MD, −9.40; 95% CI, −10.99 to −7.81; P<0.00001) in group A (10.0±2.5 days) than in group B (19.4±5.2 days). The mean VAS score 7 days after surgery in group A was significantly lower than that of group B (MD, −2.30; 95% CI, −2.80 to −1.80; P<0.00001). The length of the hospital stay in group A was significantly shorter than that in group B (MD, −7.50; 95% CI, −8.74 to −6.26; P<0.00001).

All the patients enrolled were followed up for at least 4 weeks following surgery, and there were no clinical complications in either group during this period.

## Discussion

Since skin is normally colonized by a range of microorganisms that may cause infection, defining an SSI requires evidence of clinical signs and symptoms of infection rather than microbiological evidence alone. Frequently, SSIs only affect superficial tissues, but certain more serious infections affect the deeper tissues or other parts of the body or organs manipulated during the procedure. The majority of SSIs become apparent within 30 days of an operative procedure, and most often between the fifth and tenth day. However, where a prosthetic implant is employed, SSIs affecting the deeper organs or tissues may occur several months or more after surgery (8,9). Under this context, our study included SSI patients who were more than 30 days after the primary surgery and excluded patients with any prosthetic implant.

Surveillance of SSI provides data that does not inform but influences clinical practice to decrease the risk of SSI, as well as communicating the risks of infection to patients more clearly. Since certain SSIs may take several days or longer to develop, signs of infection may not become apparent until after the patient has been discharged from hospital (10). Surveillance focused on detecting SSI during the inpatient stay is thus likely to underestimate the true rate of SSI. This issue is exacerbated by the increasing trend towards shorter lengths of postoperative hospital stay (11).

For various types of surgery, it is known that the risk of SSI is affected by the specific site of the surgery; for example, laminectomy at the cervical vertebrae is associated with a lower risk of SSI than laminectomy performed at other levels (OR, 6.7; 95% CI, 1.4 to 33.3) (12). The complexity and duration of the procedure are also indicated as risk factors of SSI. Studies of general and vascular surgery estimated that there was a two- to three-fold increased risk of SSI with increasing surgical complexity, measured as work relative value units (13–15). These studies noted that particular attention should be paid to operative patients with high risk factors of SSI. Once SSI is recognized, it should be treated promptly and effectively. Conventional treatment of SSI includes changing dressings, administration of antibiotics, removal of sutures, drainage of pus and irrigation of the wound with saline. Minor infections may respond sensitively to these treatments; however, serious cases of SSI may become exacerbated.

In serious SSI cases, the commonly used strategy is re-operation to open and clean the wound with sterile saline, but this is not always effective. Experts and researchers are making good progress in exploring novel and effective approaches for the treatment of SSI. In the present trial, an endoscopy procedure was employed for the treatment of SSI. The endoscopy procedure is in widespread use in the field of surgery and is a procedure favored by many surgeons. The procedure has advantages including minimal invasion, low risk and high cost-effectiveness. However, there are few studies in the literature reporting this minimally invasive procedure as a treatment for SSI.

The purpose of this randomized trial was to examine the clinical effectiveness of using endoscopy for the treatment of SSI in wound healing. Unlike in conventional surgery for SSI, we were able to completely remove the necrotic tissue and infected suture in visualization under the endoscopy procedure. For small sinuses that could not be reached by endoscopy, the necrosis was removed by biopsy clip (16,17). The results demonstrated that endoscopy decreased the wound healing time, the rate of skin transplantation, the blood loss and the length of hospital stay. Subsequently, the cost of treatment for the endoscopy group was significantly lower than that for the conventional surgery group. In addition, the patients in group A experienced less pain than those in group B. During the follow-up period, there were no clinical complications in either group.

In conclusion, the present study demonstrated that the endoscopy procedure for the treatment of SSI reduces the wound healing time compared with traditional surgery, without increasing the risk of any clinical events. Endoscopy was not only effective but also safe in the therapy of serious SSI. However, a further randomized control trial is necessary to determine the effectiveness and safety for the treatment of serious SSI.

## Figures and Tables

**Table I:** Baseline characteristics of the two groups of patients.

Characteristics	Group A	Group B	P-value
Number of patients	57	49	
Age (years)	35.5±5.5	34.8±7.2	0.95
Male:female ratio	37:20	30:19	0.90
Body mass index	30.4±3.2	28.3±4.4	0.87
Primary surgery			0.76
Chest surgery	5	3	
Abdomen surgery	33	27	
Limb surgery	7	7	
Lumbar surgery	8	6	
Other	4	6	
Size of infected site, cm			0.67
≤5	10	8	
5–10	26	23	
10–15	13	10	
≥15	8	8	

**Table II:** Outcomes of the treatment groups.

Outcomes	Group A	Group B	MD and 95% CI	P-value
Duration of surgery (min)	128±56	104±72	24.00 (−0.85 to 48.85)	0.06
Blood loss (ml)	327±89	405±177	−78.00 (−132.68 to −23.32)	0.005
Wound healing (days)	10.0±2.5	19.4±5.2	−9.40 (−10.99 to −7.81)	<0.00001
Rate of skin transplantation	3/57	6/49	0.40 (0.09 to 1.68)	0.21
VAS 7 days post-surgery	3.2±1.5	5.5±1.1	−2.30 (−2.80 to −1.80)	<0.00001
Saline volume of irrigation (ml)	3880±1205	4650±909	−770.00 (−1082.82 to −457.18)	<0.00001
Length of hospital stay (days)	15±4.1	22.5±2.3	−7.50 (−8.74 to −6.26)	<0.00001

MD, mean difference; VAS, visual analog scale (1–10 points).
